# Survival of outbreak, food, and environmental strains of *Listeria monocytogenes* on whole apples as affected by cultivar and wax coating

**DOI:** 10.1038/s41598-019-48597-0

**Published:** 2019-08-21

**Authors:** Dumitru Macarisin, Ishani Sheth, Minji Hur, Anna Wooten, Hee Jin Kwon, Zhujun Gao, Antonio De Jesus, Wayne Jurick, Yi Chen

**Affiliations:** 1 Office of Regulatory Science, Center for Food Safety and Applied Nutrition, Food and Drug Administration, College Park, MD USA; 2 Food Quality Laboratory, Agricultural Research Service, United States Department of Agriculture, Beltsville, MD USA

**Keywords:** Applied microbiology, Pathogens

## Abstract

The 2014–2015 U.S. nationwide outbreak of listeriosis linked to apples used in commercially produced, prepackaged caramel apples was the first implication of whole apples in outbreaks of foodborne illnesses. Two case patients of this outbreak didn’t consume caramel apples but did eat whole apples, suggesting that contaminated whole apple may serve as a vehicle for foodborne listeriosis. The current study evaluated the effect of conventional fruit coating with wax and that of apple cultivar on the survival of outbreak-associated and non-outbreak *Listeria monocytogenes* strains on *Red Delicious*, *Granny Smith* and *Fuji* apples during 160 days under simulated commercial storage. *L*. *monocytogenes* survived in calyxes and stem ends of apples of all 3 cultivars through the duration of the experiment. After 2 months of storage, significantly (p < 0.05) larger *L*. *monocytogenes* populations were recovered from apples coated with wax than those un-waxed, regardless of the cultivar. No differences in survival amongst *L*. *monocytogenes* strains (serotypes 1/2a and 4b) from clinical, food, and environmental sources were observed. The observation that coating with wax facilitates prolonged survival of *L*. *monocytogenes* on whole apples is novel and reveals gaps in understanding of microbiological risks associated with postharvest practices of tree fruit production.

## Introduction

To date, two multistate outbreaks of *Listeria monocytogenes* infections were associated with the consumption of caramel apples. In the recent U.S. multistate listeriosis outbreak in 2017, three illnesses were caused by indistinguishable *L*. *monocytogenes* strains and the epidemiological investigation revealed that caramel apples were the likely source of the outbreak^1^. The earlier multinational outbreak of listeriosis in the U.S. and Canada in 2014–2015 was linked to whole apples used in commercially produced caramel apples and resulted in 35 illnesses and 7 deaths^2^. Thirty-one of the outbreak case patients reported eating commercially produced, prepackaged caramel apples before becoming ill^2^. The follow-up investigation determined that all implicated caramel apples were prepared using fruits from a single grower (Producer A) and demonstrated that fresh whole apples were the contaminated ingredient^3^. *L*. *monocytogenes* was also isolated from whole apples distributed in retail by Producer A and collected at various points in the distribution chain^3^.

Following the 2014–2015 caramel apple outbreak much of the research efforts were focused on understanding how *L*. *monocytogenes* in caramel apples could reach levels that were of significant risk to humans^4,5^. However, 3 of the case patients of this outbreak did not consume caramel apples, and actually ate fresh cut (one patient) or whole (2 patients) apples^3^, which suggested that *L*. *monocytogenes* from whole apples could cause human illnesses. On the other hand, whole apples were reported not to support *L*. *monocytogenes* proliferation under room temperature storage (up to 2 weeks) and refrigerated storage (up to 3 months)^5,6^. The shelf life of whole apples is very long compared to other ready-to-eat fresh produce and fresh apples are preserved up to 12 months without significant decline in organoleptic qualities^7^. Thus, fresh apples often reach the consumer 6–12 months after the production date, and thus, the ability of *L*. *monocytogenes* to survive on whole apples stored for more than 3 months is of great value in order to understand the risk associated with apple contamination by this pathogen, and this has not been previously evaluated. A recent study showed a decline in numbers but prolonged survival of *Listeria innocua* on whole apples during 30-week cold storage^8^. However, considering the differences in physiology, cold tolerance and stress response between *L*. *monocytogenes* and *L*. *innocua*^9–17^, we may not readily assume that the behavior of *L*. *innocua* on apples during prolonged storage reflects that of *L*. *monocytogenes*.

Apples, due to their high acidity (pH < 4.0), constitute an unfavorable environment for *L*. *monocytogenes* proliferation, thus it is possible that the strain(s) involved the 2014–2015 caramel apple outbreak may be uniquely adapted to survive on whole apples, and this highlights the need for studying outbreak-related *L*. *monocytogenes* genotypes. Therefore, it was important to evaluate the behavior of *L*. *monocytogenes* strains involved in the 2014–2015 caramel apple outbreak and those from other outbreaks, fruits, and environmental sources, on apples during prolonged (>3 months) storage.

Upon arrival at packing facilities, apples are routinely submerged in water while being sized or sorted by flume systems or washed in dump tanks^18^. The incidence of *L*. *monocytogenes* on apples prior to harvest (in the orchards) was reported to be relatively low (<1%), nevertheless carrying a potential risk of contaminating the wash water or packing facilities in general^19^. Although flume and wash waters are routinely chlorinated, the amount of available active chlorine in these conditions may fluctuate during operations due to an increasing concentration of organic matter (capable of chelating chlorine radicals) and change in the pH. To ensure an adequate level of chlorine in wash water, a continuous monitoring system coupled with automatic injection is necessary; however, it may not be available in all packing facilities. Therefore, fruit washing in inadequately sanitized water might provide conditions for cross-contamination of internal apple fruit surfaces, such as calyxes and stem cavities. Indeed, fruit washing in experimentally contaminated water was shown to facilitate infiltration of human pathogens into the apple calyx and even apple core^18,20^. Chlorine solution at the concentration as high as 2000 mg/l was ineffective in inactivating *Escherichia coli* O157:H7 that infiltrated into apple calyxes^20^. Fatemi *et al*.^21^ showed the inability of sodium iodine solution to infiltrate closed apple calyxes thereby explaining low efficiency of decontamination treatments against *Escherichia coli* O157:H7 in apples and suggesting that morphological differences among fruits may influence the survival of pathogens in apple sinuses. The extent of apple sinus openness varies substantially among cultivars^22^. *Red Delicious*, *Granny Smith* and *Fuji* apples are amongst the most popular cultivars on the domestic market, with substantial differences in both morphology and flesh (exocarp) pH^7,22,23^. The effect of cultivar-associated morphology of the fruit on the survival of *L*. *monocytogenes* on apples of different cultivars during prolonged (>3 months) storage has not been elucidated.

Another factor that we hypothesize could affect the behavior of *L*. *monocytogenes* on fresh apples is waxing, which is a common practice in the apple fruit industry. Harvesting, sorting, and washing of apples impact the natural waxy cuticle that is associated with the glossy appearance and resistance against plant pathogens, and thus create the need for coating (waxing) of whole fruits intended for retail. The application of wax coating to apples is a standard practice in the United States and many other countries^24^. In addition to the recuperation and enhancement of glossy appearance, commercial waxing of apples also dramatically extends shelf life by reducing the transpiration (water loss), slowing fruit metabolism, and delaying the release of ethylene. In the United States, shellac-based waxes are primarily used for coating apples intended for the domestic market^24^. Considering that almost all apples from conventional production are waxed (over 90% of the marketplace) and consumers eat predominantly waxed apples, this commonly employed practice should also be evaluated from the food safety prospective. Specifically, studies aiming to understand the behavior of foodborne bacteria on contaminated fruits should be designed to best simulate fruits produced in real-world conditions, and thus common conventional post-harvest treatments of fruits should be incorporated. Therefore, the data on *L*. *monocytogenes* survival on apples obtained in a recent study by Sheng *et al*.^6^, using only un-waxed apples, did not allow us to fully assess the risks associated with *L*. *monocytogenes* contamination of conventionally produced apples in commerce.

A previous study showed that waxing resulted in a dramatic reduction in *E*. *coli* O157:H7 and *Salmonella* Muenchen populations on apples^25^. However, recent sanitation monitoring, with focus on *Listeria* spp., revealed that in some apple packing facilities the occurrence of *Listeria* spp. is higher in fruit waxing areas than other areas of the packing lines (annual Mid-Atlantic Fruit and Vegetable Convention, Communication with industry), suggesting that wax residue is associated with an increased *Listeria* spp. persistence on food-contact and non-contact surfaces. Thus, we hypothesized that, if apples are contaminated by *L*. *monocytogenes*, wax coating may also facilitate the survival of this pathogen on whole fruit during subsequent long-term storage. Hence, the impact of wax application on *L*. *monocytogenes* survival on whole apples needs to be elucidated.

The objectives of the current study were: (A) to determine the effect of apple cultivar on the survival of *L*. *monocytogenes* in apple calyxes and stem areas during prolonged (160 days) cold storage; (B) to determine if apple coating with wax can facilitate the survival of *L*. *monocytogenes* on whole apples during long-term (<5 months) storage; (C) to compare the survival of *L*. *monocytogenes* strains from the 2014–2015 caramel apple outbreak, other outbreaks and tree fruit production environments on apples during prolonged cold storage. These objectives required high confidence in quantification of bacteria numbers on apples, whereas the enumeration of *L*. *monocytogenes* in food, especially at low levels, has always been challenging. The use of most probable number (MPN) method alone or in combination with direct plating on *L*. *monocytogenes*-selective chromogenic agars has shown to greatly improve the accuracy and precision of the enumeration^26,27^. Counting low levels of *L*. *monocytogenes* on fruit is especially challenging, due to very large fruit-to-fruit variations^5,28^, and thus a high number of biological replicates was utilized to enhance statistical confidence in our data.

## Materials and Methods

### Apple fruits

Washed and un-waxed organic *Red Delicious*, *Granny Smith* and *Fuji* apples were purchased from a local grocery store. The apples were of uniform size and shape and were determined to be free of visible defects, such as bruises, insect damage, cuts, and abrasions. The apples were stored at 2 °C until used.

### *L*. *monocytogenes* strains and inoculum preparation

A cocktail of 6 *L*. *monocytogenes* strains of environmental, food, and clinical origin (Supp. Table S1) was used to inoculate the apples. Fresh stock cultures of each *L*. *monocytogenes* strain were prepared as described elsewhere^29^. *L*. *monocytogenes* populations in the cultures of each individual strain were determined by spiral plating serial dilutions on RAPID’*L*.*mono* agars (BioRad, Hercules, CA) in triplicates. Cultures from each individual strain were combined to attain a 10^6^ CFU/ml six-strain cocktail of *L*. *monocytogenes*. *L*. *monocytogenes* population in the six-strain cocktail was determined by spiral plating serial dilutions on RAPID’*L*.*mono* agars in triplicates.

### Spot inoculation of apples and subsequent fruit waxing

Two hundred fruits per cultivar of *Red Delicious*, *Granny Smith* and *Fuji* apples were individually inoculated in the calyx and stem areas with the six-strain cocktail of *L*. *monocytogenes*. Prior to inoculation, the apples were placed on polyethylene backed benchcoat paper (GE Healthcare, Buckinghamshire, UK) with stem ends up and allowed to equilibrate to room temperature (RT, 23 °C) for 4 h. One hundred µl of bacterial inoculum (10^6^ CFU/ml) was applied slowly around the stem end of each fruit to simulate the quantity of wash water that may accumulate on the stem area following a postharvest rinse. The inoculated apples were left for 1 h in a laminar flow hood at RT. All apples were then turned with blossom ends up and calyxes were spot inoculated and dried in the same manner as the stem ends, thus resulting in a total inoculum of 2 × 10^5^ CFU per apple fruit.

Inoculated apples were divided in two groups using a random sampling approach^30^ and one group (100 fruits per cultivar) was subjected to waxing. Shield-Brite® AP-40 wax (Pace International, Wapato, WA) was used at full strength, as recommended by the manufacturer. Wax was applied at room temperature as a fine mist using a spray bottle as described in the literature^25^. Specifically, each apple was sprayed with five trigger pulls, one pull each was applied to the stem and calyx ends and three pulls to coat the rest of the apple. Apples were dried at RT for 30 min. Inoculated fruits, both waxed and un-waxed, were transferred in commercial type cartons lined with paper trays and placed in a cold room at 3 °C.

The inoculation of apples and waxing of half of the inoculated apples resulted in 6 treatment groups: *Granny Smith* + *L*. *monocytogenes* (I), *Red Delicious* + *L*. *monocytogenes* (II), *Fuji* + *L*. *monocytogenes* (III), *Granny Smith* + *L*. *monocytogenes* + Wax (IV), *Red Delicious* + *L*. *monocytogenes* + Wax (V), *Fuji* + *L*. *monocytogenes* + Wax (VI) (Supp. Fig. S1). To determine the effect of waxing on *L*. *monocytogenes* survival, *L*. *monocytogenes* populations recovered from waxed and un-waxed fruits of the same cultivar were compared (black arrows, Supp. Fig. S1). To evaluate the effect of cultivar on *L*. *monocytogenes* survival, *L*. *monocytogenes* populations recovered from un-waxed *Red Delicious*, *Granny Smith* and *Fuji* apples were compared and, separately, *L*. *monocytogenes* populations from waxed *Red Delicious*, *Granny Smith* and *Fuji* apples were compared (red arrows, Supp. Fig. S1).

### Enumeration of *L*. *monocytogenes* in stem areas and calyxes of inoculated apples

*L*. *monocytogenes* populations on intact apples were enumerated on days 0, 1, 3, 7, 16, 31, 62, 93 and 160. Ten waxed and un-waxed apples per cultivar were tested at each sampling interval. Using a sterile cork borer (11 mm diameter) tissue from the stem and calyx areas was removed (in radius of 5.5 mm around stem and calyx and approximately 1.5 cm depth). For each apple, stem and calyx portions were combined, weighed and then homogenized (Polytron PT2500E Homogenizer, Kinematica, Bohemia, NY) in Buffered *Listeria* Enrichment Broth (BLEB) (1:10) and 200 µl of the tissue-broth mixture was then spread plated onto two RAPID’*L*.*mono* plates. This direct plating scheme had a lower limit of detection (LOD) of 25 CFU/apple. A subset of presumptive colonies of *L*. *monocytogenes* was confirmed by real time PCR and/or API^®^ *Listeria* (bioMérieux Inc. St. Louis, MO) as described in the FDA BAM^31^. The remainder of the tissue-broth mixture was incubated as described in the FDA BAM to determine if *L*. *monocytogenes* was present in any of the samples that were negative by direct plating (i.e. below the LOD of direct plating).

When levels of *L*. *monocytogenes* were expected to be consistently above the LOD of direct plating, the enumeration method, described above, was performed. When levels of *L*. *monocytogenes* were expected to be near the LOD of direct plating, most probable number (MPN) and direct plating were performed together. When levels of *L*. *monocytogenes* were expected to be consistently lower than the LOD of direct plating, MPN was used for enumeration. After observing a decline in the level of *L*. *monocytogenes* during the experiment, on day 62 after inoculation, the enumeration of *L*. *monocytogenes* in apples was conducted using both direct plating and MPN analysis. The MPN scheme (8 tubes of 0.4 g, 8 tubes of 0.04 g, 8 tubes of 0.004 g) with a LOD of 0.03 MPN/apple was best suited for the levels of *L*. *monocytogenes* in these samples and provided a balance between labor intensity and confidence interval. MPN was calculated according to Chapter 10 of the FDA BAM^31^ and confidence intervals were derived from the method from Fisher^32^ as reported by Hurley and Roscoe^33^. The sample was homogenized as described above and then serially diluted (1:10) in BLEB. MPN tubes were then prepared and incubated at 30 °C. Selective supplements (acriflavin hydrochloride 10 mg/l, nalidixic acid, 40 mg/l and cycloheximide, 40 mg/l, Cat. SR0149, Oxoid, UK) were added after 4 h and then incubation continued for an additional 44 h. Enrichments were subsequently streaked onto RAPID’*L*. *mono* agar plates, which were subsequently incubated at 37 °C for up to 48 h. The presence of typical *L*. *monocytogenes* colonies on an agar plate was deemed positive for that corresponding MPN tube. A subset of presumptive colonies of *L*. *monocytogenes* was confirmed as described above. Enumeration of *L*. *monocytogenes* on apples on day 93 and 160 was conducted using only MPN.

### Assessing the survival of each of the inoculating strain after 3 months of storage; and detection of each inoculating serotype after 5 months of storage

At 3 months after inoculation a random set of 30 *L*. *monocytogenes* colonies were collected from RAPID’*L*. *mono* agars representing each individual treatment/cultivar combination. A total of 180 *L*. *monocytogenes* isolates (3 cultivars × 2 treatments [waxed vs. un-waxed] × 30 colonies) were subjected to whole genome sequencing (WGS). DNA was isolated from pure cultures using the Qiagen DNeasy blood and tissue kit (catalog no. 69582; Qiagen, Inc., Valencia, CA). Sequencing libraries were prepared using the Nextera XT sample preparation kit (catalog no. FC-131-1024; Illumina, Inc.), and WGS was performed using a MiSeq (Illumina, Inc., San Diego, CA) with the version 2 kit (2 × 250 bp), according to the manufacturer’s instructions. Core genome multilocus sequence analysis^34^ was performed to compare the colonies on agar plates to the original strains in the inoculation cocktail. Among the six strains, the stone fruit outbreak strain and one of the caramel apple outbreak strains belonged to singleton ST382, a newly emerged clone with limited divergence^34^; however, there was still sufficient difference between the two genomes such that they can be easily distinguished by WGS, as previously demonstrated^34^.

At 160 days after inoculation, a random set of 180 *L*. *monocytogenes* colonies were collected from RAPID’*L*.*mono* agars representing 6 treatment/cultivar combinations and subjected to multiplex PCR serotyping as described by Doumith *et al*.^35^. This multiplex PCR serotyping allows separation of 4 major *L*. *monocytogenes* serotypes, 1/2a, 1/2b, 1/2c, and 4b and was employed as a less costly alternative (comparing to WGS) for the identification of *L*. *monocytogenes* serotypes inoculated on apples.

### Statistical analysis

*L*. *monocytogenes* populations were transformed to log_10_ CFU/apple and log_10_ MPN/apple. Some samples were positive for *L*. *monocytogenes* after enrichment, but the levels of *L*. *monocytogenes* were below the LOD (25 CFU/apple) for direct plating enumeration. For statistical analysis, the level of *L*. *monocytogenes* in these samples was inferred as half of the LOD (12.5 CFU/apple). To test the resulting data for normality, the Shapiro-Wilk Normality test was applied to the data sets^36^.

Analysis of Variance (ANOVA) and one-tailed Student’s t test post hoc analysis of independent data sets for the corresponding time points was conducted on the log_10_ (CFU and MPN) values to compare the levels of *L*. *monocytogenes* recovered from waxed and un-waxed apples at various time points. Additionally, *L*. *monocytogenes* levels recovered from apples were analyzed using ANOVA to determine the effect of cultivar. If differences among cultivars were significant (p < 0.05) via ANOVA analysis, then a one-tailed Student’s t test post hoc analysis of independent data sets for the corresponding time points was conducted with a Bonferroni correction for the significance threshold of p < 0.0167.

To evaluate the agreement between direct plating and MPN, the ordinary least squares regression analysis was applied to the paired MPN and direct plating values obtained on day 62 after inoculation.

## Results

### Survival of *L*. *monocytogenes* in stem areas and calyxes of *Granny Smith*, *Red Delicious* and *Fuji* apples for 160 days as affected by cultivar

Immediately after inoculation and drying (day 0) levels of *L*. *monocytogenes* in un-waxed apples were 3.85 ± 0.09 (average ± standard error) log CFU/apple in *Granny Smith*, 3.99 ± 0.07 log CFU/fruit in *Red Delicious*, and 5.13 ± 0.06 log CFU/apple in *Fuji* (Table 1). *L*. *monocytogenes* levels in un-waxed apples gradually declined within two months after inoculation and in some apples were approaching the LOD of direct plating enumeration. Immediately after the application of wax on inoculated apples, populations of *L*. *monocytogenes* were 3.39 ± 0.09, 2.94 ± 0.12 and 3.92 ± 0.15 log CFU/apple in *Granny Smith*, *Red Delicious* and *Fuji* apples, respectively (Table 2). *L*. *monocytogenes* levels in waxed apples also gradually declined with time, and 2 months after inoculation average *L*. *monocytogenes* levels in waxed apples were in the range of 2.08 to 2.36 log CFU/apple (Table 2). *L*. *monocytogenes* levels in un-waxed apples continued to gradually decline after 2 months of storage and on day 160 were 0.62 ± 0.50, 0.41 ± 0.30 and 1.30 ± 0.42 log MPN/apple in *Granny Smith*, *Red Delicious* and *Fuji* apples, respectively (Table 1). In waxed apples of all 3 cultivars *L*. *monocytogenes* levels ceased to decline after 2 months of storage and were 2.38 ± 0.24, 2.58 ± 0.17 and 2.43 ± 0.11 log MPN/apple on day 160 in *Granny Smith*, *Red Delicious* and *Fuji* apples, respectively (Table 2).

**Table 1:** The effect of apple cultivar on *Listeria monocytogenes* survival on un-waxed apples.

Enumeration Method	Cultivar	Storage time (days)								
		0	1	3	7	16	30	62	93	160
Direct plating, log CFU/apple	*Granny Smith*	a 3.85 (0.09)	a 3.93 (0.10)	a 3.74 (0.10)	a 3.48 (0.08)	a 3.06 (0.22)	a 2.97 (0.27)	a 2.79 (0.41)		
	*Red Delicious*	a 3.99 (0.07)	ab 3.74 (0.05)	ab 3.61 (0.07)	ab 3.20 (0.14)	a 2.83 (0.21)	b 2.53 (0.17)	b 1.89 (0.15)		
	*Fuji*	b 5.13 (0.06)	ac 3.85 (0.04)	ac 3.83 (0.04)	ac 3.65 (0.05)	b 3.51(0.03)	a 3.20 (0.05)	a 2.60 (0.13)		
MPN, log CFU/apple	*Granny Smith*							2.01 (0.53) a	1.22 (0.28) a	0.62 (0.50) a
	*Red Delicious*							1.76 (0.13) ab	0.17 (0.18) b	0.41 (0.30) a
	*Fuji*							2.54 (0.14) ac	0.97 (0.20) a	1.30 (0.42) a

Values represent the averages (n = 10) and numbers in parenthesis indicate the standard error. For direct plating data, values in the same column that are preceded by a different letter are significantly (p < 0.0167) different from each other. For MPN data, values in the same column that are followed by a different letter are significantly (p < 0.0167) different from each other.

**Table 2:** The effect of apple cultivar on *Listeria monocytogenes* survival on waxed apples.

Enumeration Method	Cultivar	Storage time (days)								
		0	1	3	7	16	30	62	93	160
Direct plating, log CFU/apple	*Granny Smith*	a 3.39 (0.09)	a 2.62 (0.17)	a 2.55 (0.07)	a 2.48 (0.10)	ab 1.90 (0.20)	a 2.46 (0.31)	a 2.36 (0.23)		
	*Red Delicious*	b 2.94 (0.12)	a 2.77 (0.11)	a 2.53 (0.18)	a 2.42 (0.19)	ac 2.59 (0.15)	a 1.95 (0.19)	b 2.08 (0.21)		
	*Fuji*	c 3.92 (0.15)	b 1.83 (0.13)	a 2.45 (0.27)	a 2.47 (0.22)	a 2.20 (0.15)	a 1.91 (0.23)	a 2.33 (0.20)		
MPN, log CFU/apple	*Granny Smith*							2.32 (0.19) a	1.77 (0.34) a	2.38 (0.24) a
	*Red Delicious*							2.32 (0.14) a	1.66 (0.22) ab	2.58 (0.17) a
	*Fuji*							2.48 (0.17) a	2.42 (0.15) ac	2.43 (0.11) a

Values represent the averages (n = 10) and numbers in parenthesis indicate the standard error. For direct plating data, values in the same column that are preceded by a different letter are significantly (p < 0.0167) different from each other. For MPN data, values in the same column that are followed by a different letter are significantly (p < 0.0167) different from each other.

**Figure 1:**
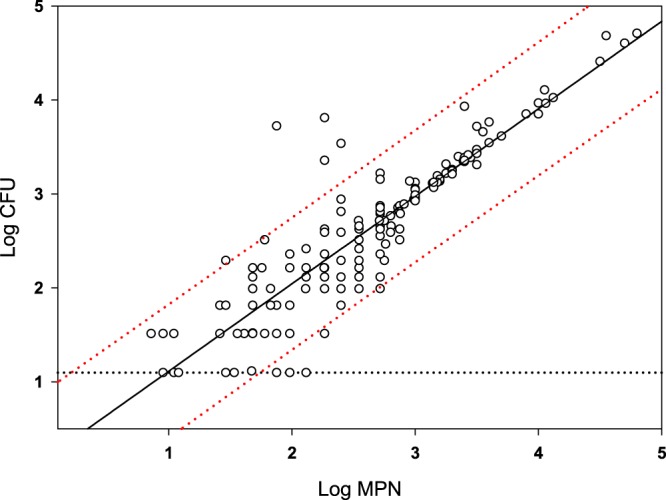
Correlation of direct plating (y) vs. MPN (x) estimates of *L*. *monocytogenes* levels in apples enumerated on day 62 after inoculation. The black line is the result from the ordinary least squares regression analysis, i.e. log_10_(*y*) = 0.9314 × log_10_(*x*) + 0.01811. The dotted red lines are the 95% prediction interval from the regression. The horizontal dashed line represents half of the limit of detection for the direct plating method.

To assure an accurate estimation of low (≤2 log CFU/apple) levels of *L*. *monocytogenes*, on day 62 after inoculation, the enumeration of *L*. *monocytogenes* in apples was concomitantly conducted by direct plating and MPN enumeration. Paired data sets on *L*. *monocytogenes* levels, obtained for all samples on day 62 (Tables 1 and 2), were used to estimate the correlation between direct plating and MPN. The scatter plot (Fig. 1) illustrates the level of agreement between the estimates provided by MPN vs. direct plating. An ordinary least squares regression of the logarithm of the MPN results (x, in MPN/apple) and the logarithm of the direct plating results (y, in CFU/apple) provided an equation of log_10_(*y*) = 0.9314 × log_10_(*x*) + 0.01811 (R^2^ = 0.81) showing a significant (p = 1.E-65) positive correlation. Because MPN enumeration correlated well with direct plating and was more suitable for estimation of low *L*. *monocytogenes* populations in foods^37^, at following sampling intervals (day 93 and 160 after inoculation) *L*. *monocytogenes* enumeration was conducted by MPN only (Tables 1 and 2).

Average *L*. *monocytogenes* populations were compared amongst *Granny Smith*, *Red Delicious* and *Fuji* apples at the same time intervals during storage to evaluate the cultivar effect. Overall, significantly lower (p < 0.0167) levels of *L*. *monocytogenes* were recovered from un-waxed *Red Delicious* compared to un-waxed *Fuji* apples at all time points except day 160 (Table 1). Average *L*. *monocytogenes* populations recovered from un-waxed *Granny Smith* apples were also greater than those from un-waxed *Red Delicious*; however, the difference was not always statistically significant.

The effect of cultivar was less evident in waxed apples, and significant (p < 0.0167) differences in *L*. *monocytogenes* levels among cultivars were only detected early in storage, on day 0, 1, 16 and 93 after inoculation (Table 2).

Interestingly, the sinuses of *Fuji* apples naturally harbored microorganisms which abundantly grew on RAPID*’L*.*mono* selective agars (Supp. Fig. S2). The analysis of these non - *L*. *monocytogenes* colonies from RAPID*’L*.*mono* agars also revealed the presence of non-pathogenic *Listeria* spp. (data not shown).

### Survival of *L*. *monocytogenes* in stem areas and calyxes of *Granny Smith*, *Red Delicious* and *Fuji* apples during 160 days as affected by wax application

**Figure 2:**
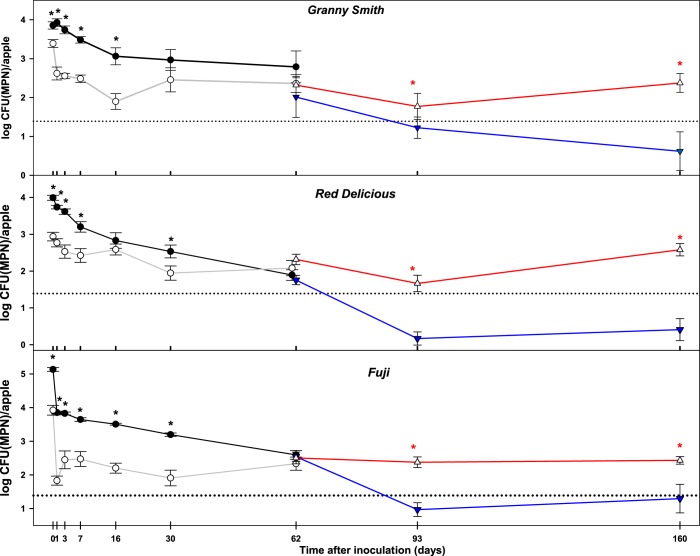
*L*. *monocytogenes* survival on *Granny Smith*, *Red Delicious* and *Fuji* apples as affected by the application of wax coating. Changes in *L*. *monocytogenes* populations in waxed (open circles, grey line) and un-waxed (black circles, black line) apples were assessed by direct plating (LOD 1.39 log CFU/apple; dotted line) before day 62 and by the MPN analysis in waxed (open triangles, red line) and in un-waxed (blue triangles, blue line) apples after day 62. Data represent the averages (*n* = 10) ± standard error. Asterisks (black for direct plating and red for MPN) indicate values that are statistically significantly different (p < 0.05) in waxed from corresponding values in un-waxed apples.

On average, *L*. *monocytogenes* levels were 0.46 to 1.21 log CFU/apple lower (p < 0.05) in waxed apples than those in un-waxed apples on day 0 (Fig. 2) which indicates that the application of wax caused an instant reduction in *L*. *monocytogenes*. During the first month after inoculation, *L*. *monocytogenes* levels in waxed apples were consistently lower (p < 0.05) than those in un-waxed apples (Fig. 2). However, this trend started to change after 2 months of storage. In un-waxed apples of all three cultivars, *L*. *monocytogenes* levels continued to gradually decline after 2 months, and on average were ≤1 log MPN/apple by the 3^rd^ month (Fig. 2). In waxed apples; however, from 2 months of the storage *L*. *monocytogenes* levels stopped declining and were approximatively 2.5 log MPN/apple at the end of the storage period (160 days; Fig. 2). After 2 months of storage, *L*. *monocytogenes* populations on waxed apples were consistently higher (p < 0.05) than those in un-waxed apples in all three cultivars evaluated (Fig. 2).

### Survival of clinical and environmental *L*. *monocytogenes* strains in apples over time

WGS analysis of 180 isolates recovered from apples 3 months after inoculation identified the presence of all strains used for the inoculation. The largest difference in prevalence observed amongst these strains was 0.33 log CFU (Supp. Table S1). The multiplex PCR serotyping of 180 isolates recovered from apples 160 days after inoculation showed the presence of 2 *L*. *monocytogenes* serovars, 1/2a and 4b, used for the inoculation in the current study.

## Discussion

The current study evaluated the survival of clinical, food, and environmental *L*. *monocytogenes* strains on whole apples during a 160-day cold storage and whether *L*. *monocytogenes* persistence on whole apples is affected by apple cultivar and a standard industry practice (i.e. apple waxing). While causing an instant pathogen reduction after application, wax coating significantly facilitated long-term survival of *L*. *monocytogenes*, and pathogen levels were dramatically higher on waxed than on un-waxed *Granny Smith*, *Red Delicious* and *Fuji* apples from the middle to the end of storage. *Granny Smith* apples, the cultivar implicated in 2014–2015 outbreak of listeriosis, did not represent a more advantageous matrix for *L*. *monocytogenes* survival compared to *Red Delicious* and *Fuji* apples, which have not been implicated in outbreaks or recalls. *L*. *monocytogenes* strains from the 2014–2015 caramel apple outbreak did not manifest greater survival fitness on whole apples compared to unrelated clinical, food and environmental *L*. *monocytogenes* strains, and no difference in competitive fitness was detected amongst representative strains of genetic lineage I and lineage II. Our study also illustrated that the employment of robust quantification techniques, such as MPN and the use of *L*. *monocytogenes*-specific agars in direct plating enumeration, and high number of biological replication (n = 10) is critical in studying the behavior of *L*. *monocytogenes* in fresh produce.

Contamination of tree fruits by human pathogens can take place at any point in the production continuum; at harvest, washing and sorting. Low efficiency of most decontamination treatments against *Escherichia coli* O157:H7 on apples^21^ suggested calyx and stem sinuses as potential harborage sites for enteric pathogens on apples particularly if contamination occurs during fruit washing operations^20,21,38,39^. This also suggests that morphological differences of the fruit among cultivars may influence the survival of pathogens in apple sinuses. Three apple cultivars, *Red Delicious*, *Fuji*, and *Granny Smith* were chosen in the current study due to their popularity and the implication of *Granny Smith* in the 2014–2015 outbreak of listeriosis. In addition, the morphological differences among the cultivars selected were essential in addressing the objectives of the current study; specifically, open versus closed apple sinus. *Fuji* has highest percentage of fruits with open sinuses (≤63%), whereas *Granny Smith* has the lowest incidence (≤1%) of open sinuses^22^, and *Red Delicious* apples have moderate incidence of open sinuses. The extent of open sinuses in *Golden Delicious* apples was suggested to be associated with *E*. *coli* O157:H7 infiltration and persistence in fruits^21^. Open calyx tube has also been associated with an increased susceptibility of apples to phytopathogens as it provides unrestricted entrance for fungal spores and bacteria^40^. Thus, we hypothesized that the extent of sinus opening may influence the persistence of *L*. *monocytogenes* in apples. In the current study, sampling at day 0 was conducted after a 2 h drying of apples following the inoculation. Because of this drying, the levels of *L*. *monocytogenes* recovered at day 0 sampling were different on different cultivars, despite inoculating the same levels of *L*. *monocytogenes*. Specifically, average *L*. *monocytogenes* populations in un-waxed *Fuji* apples were approximatively 1.1 log higher than in *Granny Smith* and *Red Delicious*. The phenomenon of instant reduction (die-off) in populations of *L*. *monocytogenes* after inoculation on whole apples has been described; 2 h drying after the inoculation led to approximatively 2 log CFU reduction of *L*. *monocytogenes* populations on *Granny Smith* and *Gala* apples^5^. In the current study the average reduction in *L*. *monocytogenes* populations after drying on Day 0 in *Granny Smith* and *Red Delicious* apples was approximatively 1.4 log and the reduction on *Fuji* apples was only 0.3 log. We observed that *Fuji* apples harbored background microorganisms in abundance that could be attributed to wider open sinuses in this cultivar, as was previously suggested^40^, and these background microorganisms could affect the survival of *L*. *monocytogenes* on apples. Epiphytic yeasts account for the majority of postharvest microbiota in apple calyxes and stem cavities^41^. Yeast epiphytes secrete extracellular hydrolytic enzymes capable of degrading cuticle and pectin polymers^42^, covering the apple epidermis, thereby accessing the nutrients necessary to sustain their population on apple surfaces that are poor in nutrients. This in turn could have created microenvironments in the calyxes and stem cavities of *Fuji* apples with more favorable conditions for *L*. *monocytogenes* survival and thus a lesser reduction in initial population than in *Granny Smith* and *Red Delicious*. With the progression of storage, *L*. *monocytogenes* survived in larger (p < 0.0167) numbers in sinuses of un-waxed *Fuji* apples than those of un-waxed *Red Delicious*. However, *L*. *monocytogenes* population level were not considerably different between *Granny Smith* (cultivar with the smallest sinus openness) and *Fuji* (Table 1), indicating that the survival of *L*. *monocytogenes* in apples cannot be solely explained by sinus openness. *L*. *monocytogenes* persistence in waxed apples was not dramatically affected by cultivar, although overall lower populations of *L*. *monocytogenes* were recovered from *Red Delicious* than from *Fuji*, and *Granny Smith* (Table 2).

There are differences in the fruit exocarp pH among *Red Delicious*, *Fuji*, and *Granny Smith* apples, however, these differences appear to be unrelated to *L*. *monocytogenes* survival in these cultivars. The lowest *L*. *monocytogenes* populations were recovered from *Red Delicious* apples which have lowest acidity (pH = 4.3) and higher *L*. *monocytogenes* populations recovered from cultivars which have higher acidity, pH 3.9 and 3.4 in *Fuji* and *Granny Smith*, respectively^23^, suggesting that other factors may play a role in survival and growth of human enteric pathogens in apples. For example, composition and concentration of lipids and volatile compounds varies significantly among apple cultivars and changes during storage^43,44^. The ability of some fruit volatiles and fatty acids to induce morphotype modification and reduced viability in a human enteric pathogen has been recently reported^45^. Over 300 volatile compounds have been identified in apple fruit^44^ primarily esters, alcohols, aldehydes, ketones and ethers with very limited information on their antibacterial potential. Evaluation of the effect of major apple volatiles and fatty acids on *L*. *monocytogenes*, and the relation to reduced survival of *L*. *monocytogenes* in *Red Delicious* fruits, warrants further investigation. Another possibility for a better *L*. *monocytogenes* survival on *Fuji* and *Granny Smith* apples could be associated with cultivar-specific properties of the fruit epidermis (skin). The epidermis of *Fuji* and *Granny Smith* apples is most resistant to breakage and mechanical wounding comparing to other cultivars^22^. Consequently, the greater force required to penetrate the skin in *Fuji* and *Granny Smith* ensured a slower moisture loss in these apple cultivars and resulted in a lesser decline of *L*. *monocytogenes* populations compared to *Red Delicious*.

The current study showed that populations of *L*. *monocytogenes* declined after inoculation in waxed and un-waxed *Granny Smith*, *Red Delicious* and *Fuji* apples (Fig. 2). The application of wax instantly caused moderate but significant reduction in *L*. *monocytogenes* populations in waxed compared to un-waxed apples. At day 0, average *L*. *monocytogenes* populations were 0.46, 1.05 and 1.21 log CFU/apple lower in waxed than in un-waxed *Granny Smith*, *Red Delicious* and *Fuji* apples, respectively. The commercial wax used in this study, Shield-Brite AP-40, contains up to 20% isopropanol, which may be the reason that caused the immediate reduction in bacterial numbers. Different reductions in *L*. *monocytogenes* levels among cultivars on day 0 could be attributed to differences in sinus openings that determined the degree of wax infiltration and coverage of calyx and stem cavity surfaces. Indeed, the highest reduction (1.21 log) was observed in *Fuji* apples, which happened to have the highest percentage of fruits with open sinuses (≤63%), whereas the lowest reduction (0.46 log) in *Granny Smith* apples, which have the lowest incidence (≤1%) of open sinuses^22^. Lower *L*. *monocytogenes* levels in waxed apples than in un-waxed apples were observed during first two months after inoculation (Fig. 2). After 2 months, *L*. *monocytogenes* populations continued to decline in un-waxed apples and conversely stopped declining in waxed fruits of all three cultivars. At day 160 after inoculation, *L*. *monocytogenes* levels were significantly higher in waxed (2.38, 2.58, 2.43 log MPN/apple) apples than in un-waxed (0.62, 0.41, 1.3 log MPN/apple) apples in *Granny Smith*, *Red Delicious* and *Fuji* cultivars respectively (Tables 1, 2). One of the major purposes of fruit coating with wax is to reduce water loss. Therefore, an enhanced long-term survival of *L*. *monocytogenes* on waxed apples discovered in the current study could be attributed to the wax-mediated moisture retention. To the best of our knowledge, there are no prior reports demonstrating that the application of wax coating can facilitate the survival of *L*. *monocytogenes* in apples during prolonged period of cold storage.

In contrast with current findings, an earlier study on the survival of Gram negative pathogens on apples, reported that the application of shellac wax on *Red Delicious* apples significantly (p ≤ 0.05) reduced *E*. *coli* O157:H7 and *Salmonella* Muenchen populations comparing to un-waxed apples stored at 2 °C during a 6- and 12-week period, respectively^25^. The substantially poorer survival of *E*. *coli* O157:H7 and *Salmonella* Muenchen on waxed apples than that of *L*. *monocytogenes* can be attributed to higher susceptibility of Gram negative bacteria to isopropanol (that is present in shellac wax) than that of Gram positive bacteria. The resistance of Gram positive bacteria to low-molecular weight alcohols (including isopropanol) was reported to be substantially higher comparing to that of Gram negative bacteria^46^. Another factor that contributed to a lower fitness/survival of *Salmonella* and *E*. *coli* comparing to *L*. *monocytogenes* on waxed apples during cold storage could be the temperature. Indeed, in the same study^25^, when inoculated and waxed apples were stored at 21 °C for 6 weeks, a moderate increase in *Salmonella* and *E*. *coli* O157:H7 populations (0.82 and 0.56 log CFU/apple, respectively) in waxed fruits was observed compared to inoculated and un-waxed controls. This demonstrates that wax application was not the only factor affecting the survival of *Salmonella* and *E*. *coli* O157:H7 in apples, and that storage temperature played an important role as well. *L*. *monocytogenes* is a notorious psychrotroph which thrives under refrigeration temperatures. Assuming that *L*. *monocytogenes*, *Salmonella* and *E*. *coli* sustained similar injures during application of wax on apple (due to isopropanol), subsequently during cold storage *L*. *monocytogenes* cells had more favorable conditions to recover from alcohol-induced injuries, comparing to *Salmonella* and *E*. *coli* O157:H7. It should also be taken into consideration that the application of wax coating on fruits has dramatic effect on fruit gas exchange. Some coatings, such as shellac wax, have very low permeability to carbon dioxide and oxygen that induces anaerobic respiration in apple tissue leading to the encapsulation of the fruit into an atmosphere of elevated carbon dioxide and ethanol^47^. Coating with shellac wax induces the highest accumulation of ethanol in apple fruit comparing to other wax coatings^47^. Greater susceptibility of Gram negative bacteria to antimicrobial properties of ethanol than that of Gram positive bacteria^46^, can also contribute to a reduced survival of *Salmonella* and *E*. *coli* on apples coated with shellac wax than that of *L*. *monocytogenes*. Interestingly, Kampf and Hollingsworth^48^ specifically observed greater reduction in populations of *Salmonella* and *E*. *coli* than that of *L*. *monocytogenes* populations after exposure to ethanol-based sanitizer. Considering the differences in the survival outcomes between Gram positive and Gram negative pathogens on waxed apples and the complexity of factors capable of differentially affect their survival, the population dynamics of major foodborne pathogens in fresh apples during prolonged storage needs to be thoroughly evaluated.

A recent study on *L*. *monocytogenes* survival in whole un-waxed apples during a 3-month storage period at 4 °C reported a reduction in pathogen levels of 0.5–1.5 and 0.8–2.0 log CFU/apple in *Granny Smith* and *Fuji*, respectively^6^. The current study showed a substantially greater decrease in *L*. *monocytogenes* levels during a 3-month cold storage; 2.25, 2.9 and 4.1 log CFU/apple, in *Granny Smith*, *Red Delicious*, and *Fuji*, respectively. This may be attributed to the difference in *L*. *monocytogenes* detection/enumeration methods utilized. In the current study, *L*. *monocytogenes* enumeration was conducted on RAPID’*L*.*mono* selective agar, which differentiates *L*. *monocytogenes* from other *Listeria* spp. Sheng *et al*.^6^ used Trypticase Soy Agar with Yeast Extract (TSAYE) for enumeration of high (≥4 log CFU/apple) and Modified Oxford (MOX) agar for enumeration of lower (<4 log CFU/apple) levels of *L*. *monocytogenes* in apple rinsates. Most of the background flora from the fruit surface could grow on TSAYE, thus making *L*. *monocytogenes* enumeration on this medium difficult. On MOX agar all *Listeria* spp. form round black colonies surrounded by black zone, making it impossible to differentiate *L*. *monocytogenes* from other *Listeria* spp. The current study and pre-harvest surveillance of apples^19^ showed the natural occurrence of *Listeria* spp. in this fruit commodity, demonstrating inappropriateness of TSAYE and MOX media for enumeration of *L*. *monocytogenes* in apple or apple rinsates. In addition, we used MPN after prolonged storage of apples, which allowed us to precisely quantify low levels of *L*. *monocytogenes* and to confirm the accuracy of the direct plating in the current study.

Whole apples, due to their acidity (pH ≤ 4.0) have been considered among the ready-to-eat foods with minimal risk of *L*. *monocytogenes* contamination. Therefore, to evaluate whether *L*. *monocytogenes* strains involved in the 2014–2015 caramel apple outbreak developed specific traits that facilitated their growth/persistence in apples, it is important to compare the survival of the strains associated with the caramel apple outbreak to environmental isolates and strains involved in unrelated incidences of foodborne listeriosis. All 6 *L*. *monocytogenes* stains (3 of serotype 1/2a and 3 of serotype 4b) used for the inoculation were detected and cultured from waxed and un-waxed apples at consecutive periods of storage and there were no clear differences in survival among different strains of serotypes 1/2a and 4b. In addition, there were no differences observed in survival within each serotype. These two serotypes represented *L*. *monocytogenes* genetic lineages I and II, which are predominant lineages of *L*. *monocytogenes* population with significant genetic and phenotypic differences between them. Thus, the finding that strains from these two lineages, used in the cocktail inoculum, did not differ in their survival is very interesting. In the future, this assessment can be expanded to more strains from each lineage, potentially representing more serotypes, to determine whether there could be serotype-specific or strain-specific differences in the survival on apples.

The 2014–2015 multistate outbreak of *L*. *monocytogenes* infections linked to caramel apples included case patients who consumed fresh whole apples, not caramel apples^3^, suggesting that whole apples, which do not support the growth of *L*. *monocytogenes*, had either served as a vehicle for foodborne listeriosis or cross-contaminated foods that can support *L*. *monocytogenes* growth. Actually, in recent listeriosis outbreaks, frozen food and fresh fruit that do not support the growth of *L*. *monocytogenes* were implicated and available information, obtained during outbreak investigation, did not suggest high levels of *L*. *monocytogenes* consumed by case patients^26,37,49^. Therefore, the demonstration that wax coating facilitated prolonged survival of *L*. *monocytogenes* in experimentally contaminated apples indicates the need for more research in evaluating the food-safety risks associated with conventional postharvest practices of fresh fruits and broadening of our current understanding of *L*. *monocytogenes* dose-response levels. This is especially a concern since several recent outbreaks were associated with hypervirulent strains^26,50–52^ and elderly and immunocompromised patients^26,37^. In the case studies of two recent outbreaks in the United States and Europe, which involved immunocompromised patients and possibly hypervirulent strains, the probability of infection after consumption of one cell of *L*. *monocytogenes* was estimated to be almost 100,000 higher than that estimated by FAO/WHO in 2004 based on epidemiologic data of patients from all susceptible population groups^53^.

### Concluding remarks

The current study demonstrated the ability of *L*. *monocytogenes* to survive in calyxes and stem ends of experimentally contaminated un-waxed apples of 3 cultivars over 5 months under simulated commercial storage conditions, with average populations declining from approximatively 3.9–5.1 log CFU/apple to 0.5–1.3 log MPN/apple. Apple coating with commercial wax significantly facilitated *L*. *monocytogenes* survival in experimentally contaminated fruits, with pathogen populations declining from approximatively 2.9–3.9 log CFU/apple to 2.4–2.6 log MPN/apple during 160-day cold storage. Waxing is widely used by the fruit industry, thus the mechanism of such enhanced survival on waxed apples may warrant further investigation. In addition, the effect of wax on the survival of *L*. *monocytogenes* on apples or other fruits represents very interesting topics of future studies. Since the application of wax could result in wax residue on food contact surfaces in fruit packing environments, the effect of wax on the pathogen persistence on food contact surfaces should be evaluated. Future studies may consider alterative or modified practices to ensure long-lasting control of foodborne pathogens in fresh fruits, for example evaluating the incorporation of natural antibacterial compounds into wax coatings.

Cultivar-associated morphological differences, such as open vs. closed sinus, did not affect the prolonged survival of *L*. *monocytogenes* during cold storage. This suggests that *Granny Smith* apples, implicated into the 2014–2015 caramel apple outbreak, did not provide more advantageous conditions for *L*. *monocytogenes* survival compared to other apple cultivars investigated.

No differences in survival amongst clinical and environmental *L*. *monocytogenes* strains of serotypes 4b and 1/2a were observed in apples, suggesting a lack of advantage in fitness of *L*. *monocytogenes* stains involved into the 2014–2015 caramel apple outbreak, at least among the strains included in the inoculum.

## Supplementary information

Supplementary Materials
