# Supplementary material for: Survival of outbreak, food, and environmental strains of Listeria monocytogenes on whole apples as affected by cultivar and wax coating
Source: Sci Rep. 2019 Aug 21;9:12170. doi: 10.1038/s41598-019-48597-0 (PMC6704171; doi:10.1038/s41598-019-48597-0)
Supplement: Supplementary file 1 — Supplementary Materials [file 41598_2019_48597_MOESM1_ESM.pdf]

Supplementary Materials

Survival of outbreak, food, and environmental strains of *Listeria monocytogenes* on whole apples as affected by cultivar and wax coating

Dumitru Macarisin[1], Ishani Sheth[1], Minji Hur[1], Anna Wooten[1], Hee Jin Kwon[1], Zhujun Gao[1], Antonio De Jesus[1], Wayne Jurick II[2], Yi Chen[1]

[1] Office of Regulatory Science, Center for Food Safety and Applied Nutrition, Food and Drug Administration, College Park, MD, USA.

[2] Food Quality Laboratory, Agricultural Research Service, United States Department of Agriculture, Beltsville, MD, USA.

Table S1. *L. monocytogenes* strains used in the study. Relative prevalence (log CFU) of each inoculating strain within the population of 180 isolates (obtained from apples after 3 months of storage) and subjected to whole genome sequencing.

Strain ID	Strain source	Outbreak	Serotype	Prevalence log CFU
SKFI1	stone fruit	NA	1/2a	1.28
LIS0087	cantaloupe packing environment	2011 U.S. cantaloupe outbreak of listeriosis	1/2a	1.40
2208	cantaloupe	NA	1/2a	1.28
26-3b	stone fruit	2014 U.S. stone fruit outbreak of listeriosis	4b	1.56
6790	human	2014 -2015 U.S. caramel apple outbreak of listeriosis	4b	1.60
6656	human	2014 -2015 U.S. caramel apple outbreak of listeriosis	4b	1.61

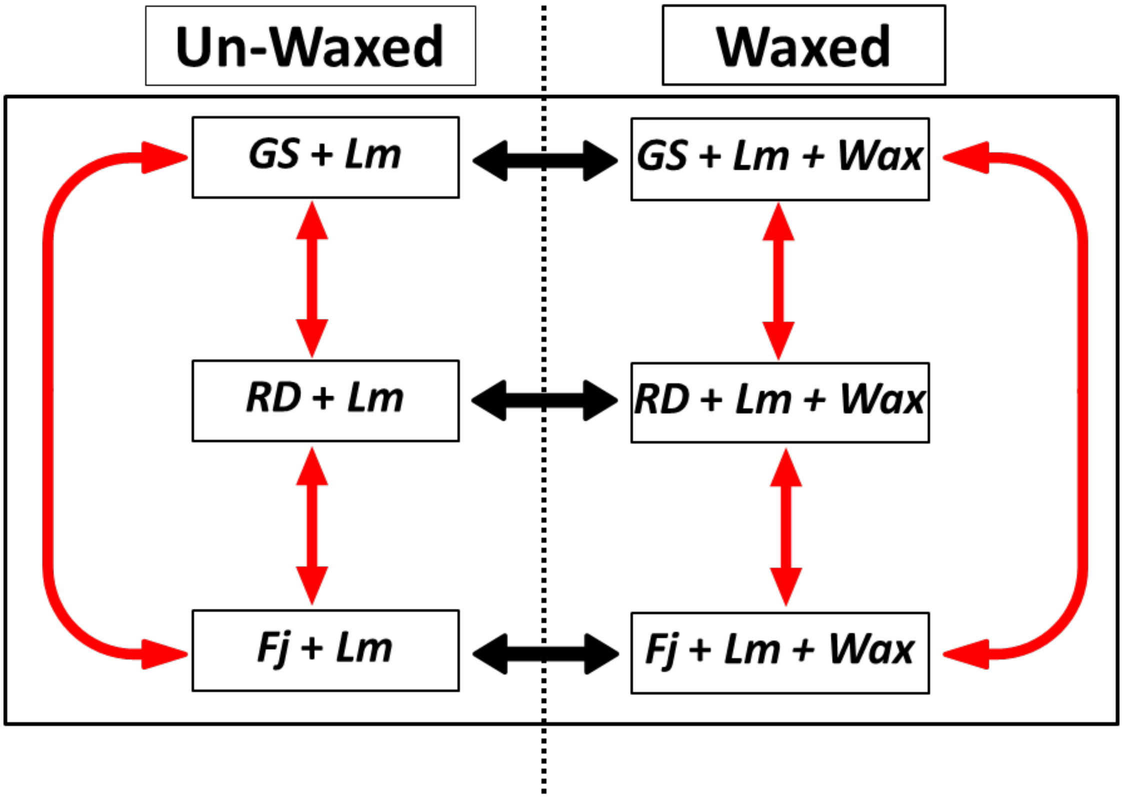

Figure S1. The schematic presentation of the experimental design with rectangles indicating the treatment groups among which *L. monocytogenes* populations were compared to determine the effect of wax coating and that of cultivar on *L. monocytogenes* survival in '*Granny Smith'* (*GS*), *'Red Delicious'* (*RD*) and '*Fuji'* (*Fj*) apples during simulated commercial storage. Apples were inoculated with *L. monocytogenes* (+ *Lm*) and after inoculation half of the fruits were coated with wax (+ Wax). At consecutive time intervals (Day 0, 1, 3, 7, 16, 31, 62, 93, and 160), *L. monocytogenes* populations recovered from waxed and un-waxed fruits were compared within a cultivar (black arrows) to determine the effect of waxing. Red arrows indicate the comparisons among *L. monocytogenes* populations recovered from apples of different cultivars. Thus, each sampling event, the effect of cultivar was evaluated by 3 comparisons amongst *L. monocytogenes* populations from waxed apples and 3 comparisons amongst *L. monocytogenes* populations from un-waxed apples.

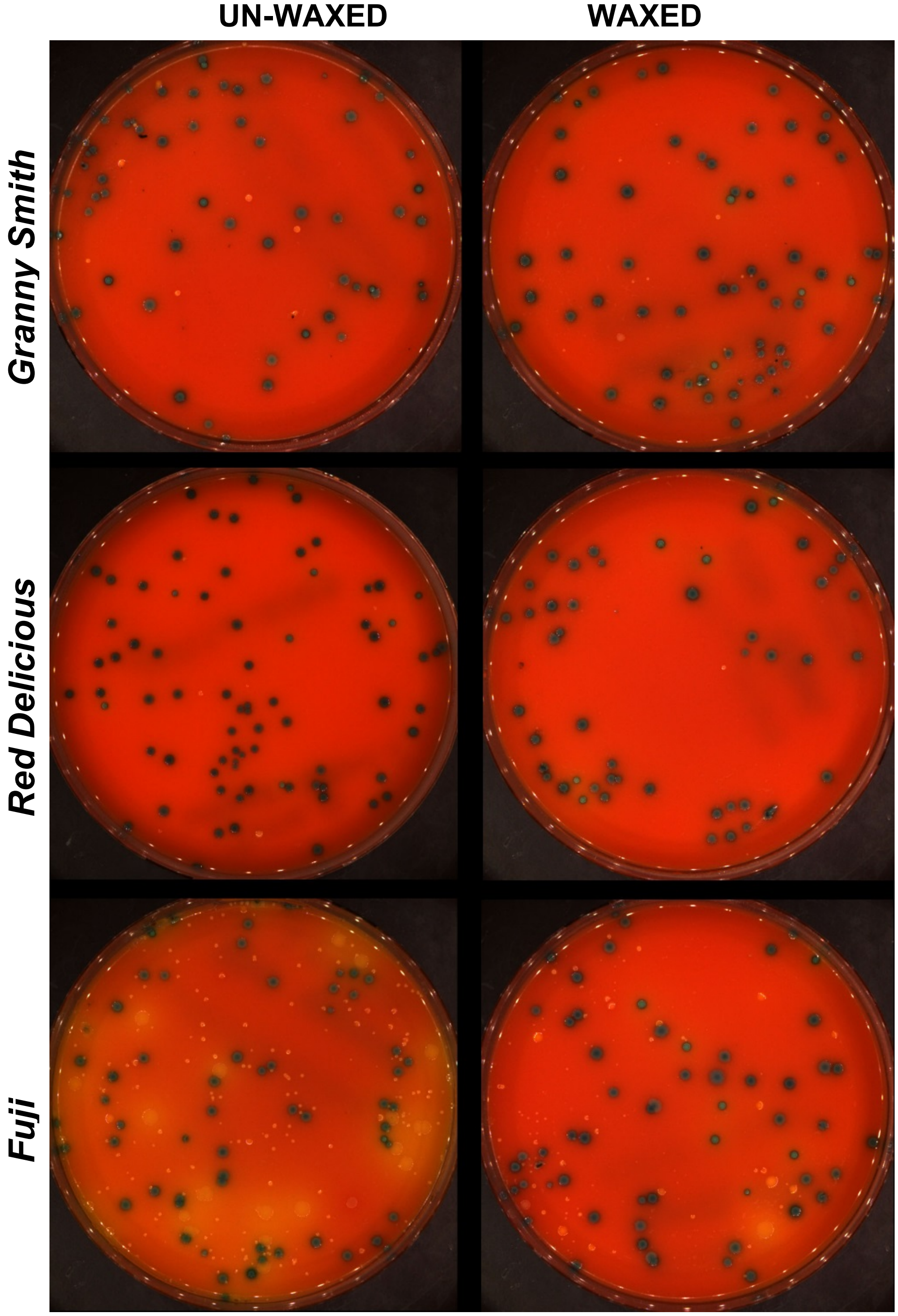

Figure S2. Images of the representative RAPID*'L.mono* agar plates (Ø =100 mm) showing *L. monocytogenes* and non - *L. monocytogenes* colonies recovered from calyces and stem areas of '*Granny Smith'*, '*Red Delicious'*, and '*Fuji*' apples.
